# Recent Advances in Antimicrobial Hydrogels Containing Metal Ions and Metals/Metal Oxide Nanoparticles

**DOI:** 10.3390/polym9120636

**Published:** 2017-11-23

**Authors:** Fazli Wahid, Cheng Zhong, Hai-Song Wang, Xiao-Hui Hu, Li-Qiang Chu

**Affiliations:** 1 Key Laboratory of Industrial Fermentation Microbiology (Ministry of Education), Tianjin University of Science and Technology, No. 29, 13th Avenue, TEDA, Tianjin 300457, China; kfazliwahid@yahoo.com (F.W.); czhong.tju@gmail.com (C.Z.); huxiaohui666666@163.com (X.-H.H.); 2 College of Chemical Engineering and Materials Science, Tianjin University of Science and Technology, No. 29, 13th Avenue, TEDA, Tianjin 300457, China; w1044674839@163.com

**Keywords:** nanocomposite hydrogels, antimicrobial activity, metal ions/nanoparticles, metal oxide nanoparticles

## Abstract

Recently, the rapid emergence of antibiotic-resistant pathogens has caused a serious health problem. Scientists respond to the threat by developing new antimicrobial materials to prevent or control infections caused by these pathogens. Polymer-based nanocomposite hydrogels are versatile materials as an alternative to conventional antimicrobial agents. Cross-linking of polymeric materials by metal ions or the combination of polymeric hydrogels with nanoparticles (metals and metal oxide) is a simple and effective approach for obtaining a multicomponent system with diverse functionalities. Several metals and metal oxides such as silver (Ag), gold (Au), zinc oxide (ZnO), copper oxide (CuO), titanium dioxide (TiO_2_) and magnesium oxide (MgO) have been loaded into hydrogels for antimicrobial applications. The incorporation of metals and metal oxide nanoparticles into hydrogels not only enhances the antimicrobial activity of hydrogels, but also improve their mechanical characteristics. Herein, we summarize recent advances in hydrogels containing metal ions, metals and metal oxide nanoparticles with potential antimicrobial properties.

## 1. Introduction

Hydrogels are three-dimensional (3D) networks formed from natural or synthetic polymers, capable of retaining a large amount of water and biological fluid in their swollen form [1]. In 1960, the first hydrogel was used for biomedical applications when poly(hydroxyethyl methacrylate) (PHEMA) was cross-linked for eye treatment [2]. Nowadays, hydrogels are widely investigated for various biomedical applications such as tissue engineering [3], drug delivery [4,5,6], cell culture [7], wound healings [8,9,10], and so on. Parallel with the developments in hydrogels and its ultimate reach to the consumer care market, many research groups have developed nanoparticles and their composites over the decades. Due to the difference in the properties of the bulk materials, nanoparticles have found promising applications in consumer products. Although nanoparticles possess novel characteristics that make them available for a vast range of applications, the questions arise regarding their safety when they come in contact with biological system [11]. One possible solution to the problem is to incorporate nanoparticles into a hydrogel matrix, resulting in decreased risk to human health and the environment. Furthermore, the innovative combination of these two completely different types of materials is considered not only to have structural diversity, but also to include a variety of properties enhancements [12]. The large spaces in the polymeric networks of hydrogels not only work as a reservoir for massive nanoparticles, but also serve as nanoreactor templates for the nucleation and growth of nanoparticles [13]. The incorporation of inorganic nanoparticles into hydrogel leads to generating new nanocomposite materials where the hydrogel can benefit from the physical (magnetic, mechanical, conducting, optical and catalytic) and biomedical (anticancer and antimicrobial) properties of the embedded nanoparticles with distinctive applications in biotechnology [14]. Therefore, various nanocomposite hydrogels have been reported for diverse applications in the biomedical field such as biosensors [15], drug delivery [16], antimicrobial [17,18] and wound healing [19].

The infectious diseases caused by pathogens such as viruses, bacteria and fungi remain a major health threat that can transform into extensive social and economic problems, even though there are major advancements in the standards of the medical technology and healthcare fields. Moreover, the misuse of conventional antibiotics has also led various pathogens to become drug-resistant [20]. To overcome such problems, there is a dwindling pipeline in the development and production of new antimicrobial agents. On the other hand, there is also an urgent need to develop new antimicrobial materials and their relevant formulation. Several classes of unique materials have emerged as substitutes of conventional antibiotics including synthetic cationic polymers [21], antimicrobial peptides [22], antimicrobial nanoparticles [23] and polymeric hydrogels. Moreover, conventional antibiotics act on a specific molecular target, such as the inhibition of protein synthesis of microbes, whereas these new materials are acting upon the entire cellular membrane by electrostatic interactions and subsequent insertion of their components into the lipid domains of the microbial membrane to generate pores. This eventually leads to cell rupture, leaking of cytoplasm and the death of cells [24,25]. Due to their great biomedical significance, hydrogels are widely researched for antimicrobial applications. In these antimicrobial hydrogels, the nanocomposite hydrogels consisting of inorganic moieties are promising candidates to inhibit bacterial growth, making them attractive in biomedical and biotechnological fields [26]. Therefore, this review article pays special attention to antimicrobial nanocomposite hydrogels. Especially, we have focused on antimicrobial hydrogels loaded with metal and metal oxide nanoparticles. There are some excellent review articles that explore antimicrobial hydrogels [25] and antimicrobial nanoparticles [27]. In this review, we have illustrated antimicrobial nanocomposite hydrogels, mainly the recent advancements for the past few years. 

## 2. Antimicrobial Hydrogels from Natural and Synthetic Polymers

Polymeric hydrogels hold remarkable potential for antimicrobial and wound healing applications. Hydrogels are polymeric networks that can be tailored to mimic the physiochemical properties of human tissues [8]. There is an excellent review explaining antimicrobial hydrogels [25]; here, we will briefly introduce some natural and synthetic polymeric hydrogels. 

### 2.1. Antimicrobial Hydrogels from Natural Polymers

Chitosan is one of the polysaccharides obtained from chitin by deacetylation reaction, which is one of the most abundant sugar-based biopolymers. Chitosan is biocompatible, biodegradable and has low toxicity; therefore, chitosan and its derivatives are researched most widely in biomedical fields [28]. Chitosan products were approved as field bandages by the U.K. and USA in the Iraq and Afghanistan wars because of their natural antimicrobial and hemostatic properties [29]. Moreover, chitosan hydrogels can be easily prepared by blending or cross-linking with suitable materials. This biopolymer is a versatile material, which can be modified for desired biomedical applications. Chitosan hydrogel for wound healing and antimicrobial applications was designed by the combination with nerolidol [30]. The hydrogelation was carried out in acidic medium with 2% or 4% nerolidol. The prepared hydrogel was tested against *S. aureus*, and the hydrogel showed complete inhibition of bacterial growth. Hydrogel with 2% nerolidol exhibited excellent healing in mice. On the seventh day of treatment, the beginning of re-epithelization and reorganization of collagen was observed. The authors claimed the potential use of the prepared hydrogel for wound healing and antibacterial applications [30]. Natural polymers have significant antimicrobial activity; however batch-to-batch disparity in the molecular weights of natural polymers may affect their physical properties. Therefore, another study on the effect of the molecular weight of natural polymers on antimicrobial performance should be carried out. Immunogenicity caused by the impurities in natural polymers is usually reported with the usage of natural polymers. This should be considered when designing natural antimicrobial hydrogels.

### 2.2. Antimicrobial Hydrogels from Synthetic Polymers

Apart from natural polymers, synthetic cationic polymers have been widely researched as important antimicrobial substitutes for conventional antibiotics. These polymers can be prepared in many ways, such as reversible addition fragmentation chain-transfer polymerization (RAFT), ring opening polymerization (ROP) and atom transfer radical polymerization (ATRP). These materials may contain biodegradable or non-biodegradable polymer backbones. In fact, due to the threat of drug-resistant pathogens, these materials have attracted tremendous research interest. A large amount of antimicrobial synthetic cationic polymers has been reported in the literature, such as poly(ethyleneimine)s [31], poly-β-lactams [32], poly(acrylate) [33], poly(norbornene) [34], poly(arylamide) [35], poly-α-amino acids [36] and polycarbonates [37]. These polymers are also reported as hydrogels for antimicrobial applications. 

## 3. Antimicrobial Activity of Metals and Metal Oxide Nanoparticles

Several metals and metal oxides such as silver (Ag), gold (Au), silver oxide (Ag_2_O), zinc oxide (ZnO), copper oxide (CuO), titanium dioxide (TiO_2_) and magnesium oxide (MgO) have been reported to exhibit marked antimicrobial performance against various microbial strains [27,38]. Therefore, these materials have attracted great interest of the healthcare and environmental industries, which are seeking novel and better agents to prevent or control microbial infections. Several studies have been carried out to explain the antimicrobial mechanism and efficacy of metals and metal oxide nanoparticles, but the existent literature is still inadequate and controversial. Nevertheless, there are two popular proposed mechanismes in this regard: (1) antimicrobial toxicity arises due to the production of metal ions by nanoparticles; (2) oxidative stress via the generation of reactive oxygen species (ROS) on the surfaces of nanoparticles [39,40,41,42]. Different ions, free radicals (such as ^•^OH, ^1^O_2_), small molecules (such as H_2_O_2_) or super oxides (O_2_^−^) are examples of highly reactive ROS species that can be generated on the surfaces of these inorganic nanoparticles and can cause bacterial cell death. ROS-induced damages and bacterial death include oxidative lesions, oxidative stress and membrane lipid peroxidation. Moreover, ROS can affect the proteins and nucleic acids of bacterial cells. For example, oxidative stress induced by Ag_2_O nanoparticles damages DNA of *E. coli*, which in turn interferes with the bacterial cell cycle and causes the death of the bacterial cell [43,44,45]. Similarly, CuO nanoparticles (CuO-NPs) have been reported to generate ROS, i.e., superoxide ions, when absorbed on the surfaces of bacterial cells; resulting in a ROS-induced bactericidal effect in both *E. coli* and *S. aureus* [46].

**Figure 1:**
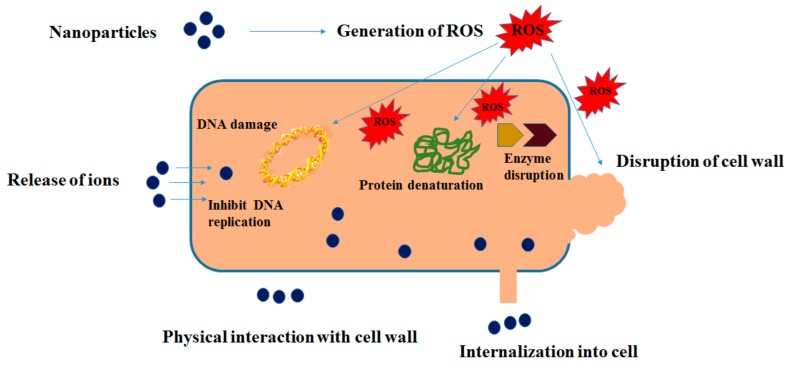
Various antibacterial activities of metal nanoparticles [27].

Moreover, physiochemical and morphological properties of nanoparticles have been demonstrated to exert an effect on their antimicrobial properties [47,48]. Small nanoparticles reveal the strongest bactericidal effect [44,49,50]. The positively-charged surfaces of metals and metal oxide nanoparticles facilitate their binding with the negatively-charged surfaces of bacterial cells, which may result in the augmentation of the bactericidal effect [47]. The shapes of nanoparticles also affect their antimicrobial activity [51,52]. Figure 1 shows the proposed antibacterial mechanism of metals and metal oxide nanoparticles. 

## 4. Designing of Nanocomposite Hydrogels

**Figure 2:**
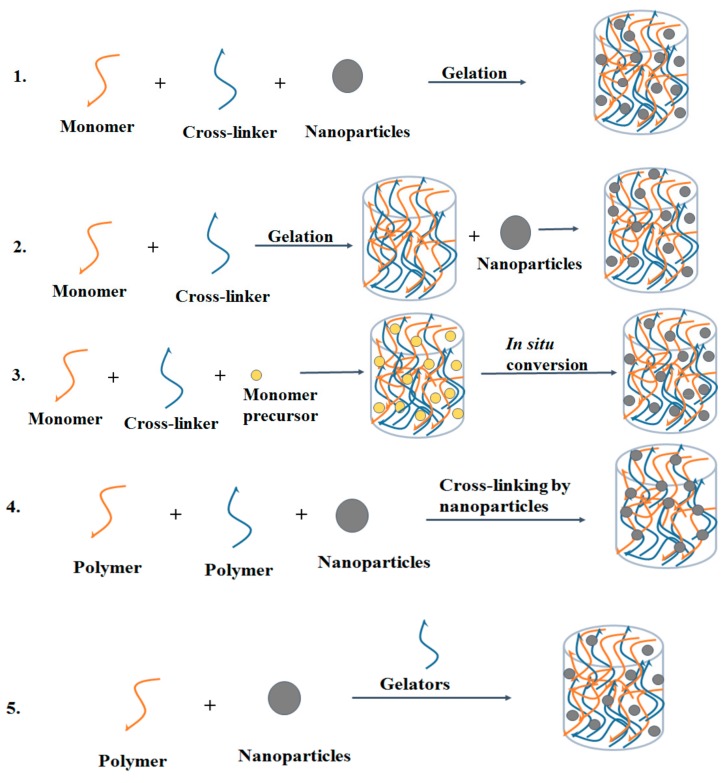
Five main approaches to get nanocomposite hydrogels with a uniform distribution of nanoparticles: (**1**) formation of the hydrogel in nanoparticle suspension; (**2**) incorporation of nanoparticles into pre-formed hydrogel; (**3**) formation of reactive nanoparticles within a pre-formed hydrogel; (**4**) cross-linking of hydrogel by nanoparticles and (**5**) hydrogel formation using nanoparticles, cross-linking agents and polymers.

A diverse range of nanocomposite hydrogels has been developed with the incorporation of varying types of nanoparticles in the hydrogel network. There are five main methods to design nanocomposite hydrogels: (1) formation of hydrogel in nanoparticle suspension, (2) incorporation of nanoparticles into pre-formed hydrogel, (3) formation of reactive nanoparticles within a pre-formed hydrogel, (4) cross-linking of the hydrogel using nanoparticles and (5) hydrogel formation using nanoparticles, cross-linking agents and polymers [12]. The schematic representation of these five approaches is illustrated in Figure 2. 

### 4.1. Formation of Hydrogel in Nanoparticle Suspension

The simplest method to develop a nanocomposite hydrogel is the hydrogelation of monomers in a suspension of nanoparticles. This method has been applied to prepare optically-responsive nanocomposite hydrogels [12]. For example, Janova and Dekany [53] synthesized poly(acrylamide)- and poly(*N*-isopropyl-acrylamide)-based hydrogel film containing gold nanoparticles. Monomers along with cross-linking agent were added to the nanoparticle suspension, and after the addition of the initiator, the polymer films were formed by photopolymerization on the surface of an interdigital microelectrode. Liu et al. [54] also used this approach to synthesize photo-modulable thermos-responsive hydrogel (acrylic-based/TiO_2_) using unilamellar titania nanosheets as photo-crosslinkers. The nanoparticles acted as photo-catalysts rather than cross-linkers, and a bifunctional monomer *N*,*N*′-methylenebisacrylamide was essential for the production of mechanically-durable hydrogels. 

### 4.2. Incorporation of Nanoparticles into Pre-Formed Hydrogel 

In this method, metal/metal oxide nanoparticles are mixed with pre-formed hydrogel, and then, the nanoparticles infiltrate into the hydrogel. Gogoi et al. [55] put chitosan hydrogel in a silver nanoparticle (Ag-NPs) suspension resulting in the adsorption of Ag-NPs in the hydrogel matrix. The inhaling of MgO nanoparticles into the hydrogel matrix has also been reported [56]. However, this method has some discrepancies including that the dispersion of metal/metal oxide nanoparticles inside the hydrogel is not easy due to the high surface charge on nanoparticles. The leaching of nanoparticles out of the hydrogel matrix occurs if the cross-linking density is low. Furthermore, the nanoparticles are physically embedded within the hydrogel matrix. This may imply the continuous release of nanoparticles from the hydrogel into external environment, leading to a toxic effect to host tissues [57].

### 4.3. Formation of Reactive Nanoparticles within a Pre-Formed Hydrogel

In this approach, a pre-formed hydrogel acts as a microreactor, in which metal/metal oxide nanoparticles are formed from its precursor, after a series of required treatments. The process is gaining popularity for its technological advantages over the ex situ method because the nanoparticle size and morphology can be controlled with relative ease [58]. The metals ions can be absorbed by the functional group of hydrogel by electrostatic or dipole ion interaction, and metal ions would form metal/metal oxide nanoparticles by means of self-assembly [59], electrochemical deposition, *co*-precipitation [60], etc. During the absorption, the ion exchange occurs between metal ions present in solution and the functional group present within the hydrogel. A high rate of absorption capacity is much better for the synthesis of metal/metal oxide nanoparticles. After absorption, metal ions can be oxidized to metal oxide nanoparticles with ammonium hydroxide or reduced to metal nanoparticles by reducing agents such as NaBH_4_ [61], H_2_ [62], citrate [63], etc., which contribute to different particle sizes. Natural polymer-based hydrogels such as polysaccharides are also reported to synthesize metal/metal oxides nanoparticles with the help of these chemicals [26,64].

### 4.4. Cross-Linking of Hydrogel Using Nanoparticles

Hydrogels can be cross-linked by nanoparticles such as titania, silica, graphene oxide, carbon nanotubes and nanoclay [65,66,67,68]. The organic cross-linkers usually can covalently attach a few polymer chain (i.e., less than 10) due to the limited number of free-end groups, whereas the nanoparticle cross-linkers with high surface activity have the capability to attach a large number of polymer chains (i.e., more than 100). Furthermore, there is a covalent bond in organic cross-linkers with polymer chains, while there is physical bonding (ionic interaction or coordination) in nanoparticles with polymer chains. On distortion, a part of the polymer chains can detach from the nanoparticle and partially release the stored elastic energy. Owing to the high energy dissipation, this nanocomposite hydrogel reveals higher toughness and stretchability than conventional covalently-cross-linked hydrogel, thus presenting potential for artificial muscles and cartilage applications. Wang and Gao [69] developed a constitutive model of nanocomposite hydrogel to explain its microscopic mechanics, including microarchitecture and the development of a nanoparticle cross-linked polymer chain during the mechanical distortion of hydrogel. The model enabled understanding the Mullins effect of hydrogel nanocomposites, as well as the effects of size and concentration of nanoparticles on their cyclic stress-strain behaviors. The theory was quantitatively confirmed by the tensile tests on a nanocomposite hydrogel using nanosilica as cross-linking agent. Moreover, the theory can be further extended to explain the mechanical behaviors of existing hydrogels cross-linked by nanoclay and the instability of composite hydrogels cross-linked by organic cross-linkers and nanoparticles. The model could be used to reveal the interaction of the particle-polymer chain and to design hydrogels with high mechanical strength and stretchability. 

### 4.5. Hydrogel Formation Using Nanoparticles, Polymers and Cross-Linking Agents 

Using this approach, Wu and coworkers [70] produced a three-dimensional hydrogel network consisting of Si-NP coated with polyaniline. The hydrogel framework had several positive features, i.e., porous space for volume expansion, a constant electrically conductive polyaniline network, and binding the Si surface either by electrostatic interaction with positively-charged polymer or hydrogen bonding with phytic acid. The hydrogel was formed by mixing aniline, phytic acid and Si-NP in water followed by the addition of ammonium persulfate as an oxidizer. The aniline was polymerized and cross-linked, resulting in a dark green viscous gel due to the presence of a gelator (phytic acid). Consequently, the gel was bladed onto a copper foil current collector and dried to form uniform film for electrochemical applications.

## 5. Antimicrobial Hydrogels Containing Metal Ions

The incorporation of metal atom/ions into hydrogels constitutes an irreplaceable way to introduce unique characteristics of inorganic components or metal complexes, such as catalytic, photoresponsive, photochemical, redox, conductive, as well as antibacterial properties. Along with the development of hydrogels from small organic compounds, the development of metallogels largely begins with organogels [71,72]. There are some excellent reviews for the readers to study the metallogels in details [73,74]. Here, we will focus on the metallogels for antimicrobial properties. 

Silver ions are popular antimicrobial agents because of their broad spectrum of antiviral, antibacterial and antifungal activity [75]. The antimicrobial mechanism of silver ions involves inactivation of membrane-bound proteins, causing changes in the morphology of cell, inhibition of cell replication [76,77] and the disorder of solute and electron transport systems. These can interfere with essential enzymes and DNA, reducing the production of vital cell components such as adenosine triphosphate (ATP) [77,78,79]. Silver ions can target multiple sites, which is suitable to reduce the development of drug-resistant strains. [80]. Moreover, silver ions have been reported to improve wound healing by modulating inflammatory response [81]. The antimicrobial activity of silver ions, as well as inflammatory regulation activities are of great interest to develop wound healing products. Certain products containing silver are normally used to treat Gram-positive and Gram-negative bacteria, as well as some antibiotic-resistant bacteria such as *P. aeruginosa* [77]. 

Hydrogels containing silver ions have been reported in the literature. For example, Martin et al. [82] prepared a hydrogel consisting of chitosan, tea tree oil (TTO) and Ag^+^ for wound management applications. Combining the concepts that TTO and silver ions have broad spectrum antimicrobial activity while chitosan is mucoadhesive, biocompatible and has controlled release properties, the objective of the work was to develop a hydrogel system that may behave as an antimicrobial agent and may enhance wound healing. The resulting hydrogel showed bactericidal performance against *C. albicans*, *S. aureus* and *P. aeruginosa*. The study showed the feasibility of developing hydrogels as a controlled release system of antimicrobial agents (TTO and Ag^+^ in combination) for the treatment of acute wounds. In another research work, sulfobetaine-based copolymer (SBE-DAM) was developed; using ([2-(methacryloyloxy)ethyl]dimethyl-(3-sulfopropyl), dopamine methacrylamide (DAM) and ammonium betaine by a free radical reaction [83]; which was then used as a platform for the synthesis of hydrogel in the presence of various metal ions (Ti^3+^, Fe^3+^ and Ag^+^) at different pH values. The viscoelastic measurements showed that the noncovalently cross-linked hydrogels by Ti^3+^ and Fe^3+^ revealed a similar elastic behavior as covalently cross-linked IO_4_^-^- or Ag^+^-based ones. The Ti^3+^- and Fe^3+^-based hydrogels showed reversibility, and this was confirmed by treating these hydrogels with EDTA as a strong coordinating agent. To investigate the self-healing ability of hydrogel, the hydrogel based on Ti^3+^ showed a complete self-healing property, while hydrogel based on Fe^3+^ showed 92% self-healing. The covalently-cross-linked hydrogels showed a maximum of 72% self-healing ability. The antibacterial activity of hydrogel could be improved by the amount of Ag^0^ production. The cytotoxicity measurements of SBE-DAM were carried out against the NIH/3T3 fibroblast cell line by the MTT essay and human dermal fibroblast cell (F121) by the direct method. The results showed the non-toxic behavior of developed polymer. 

**Figure 3:**
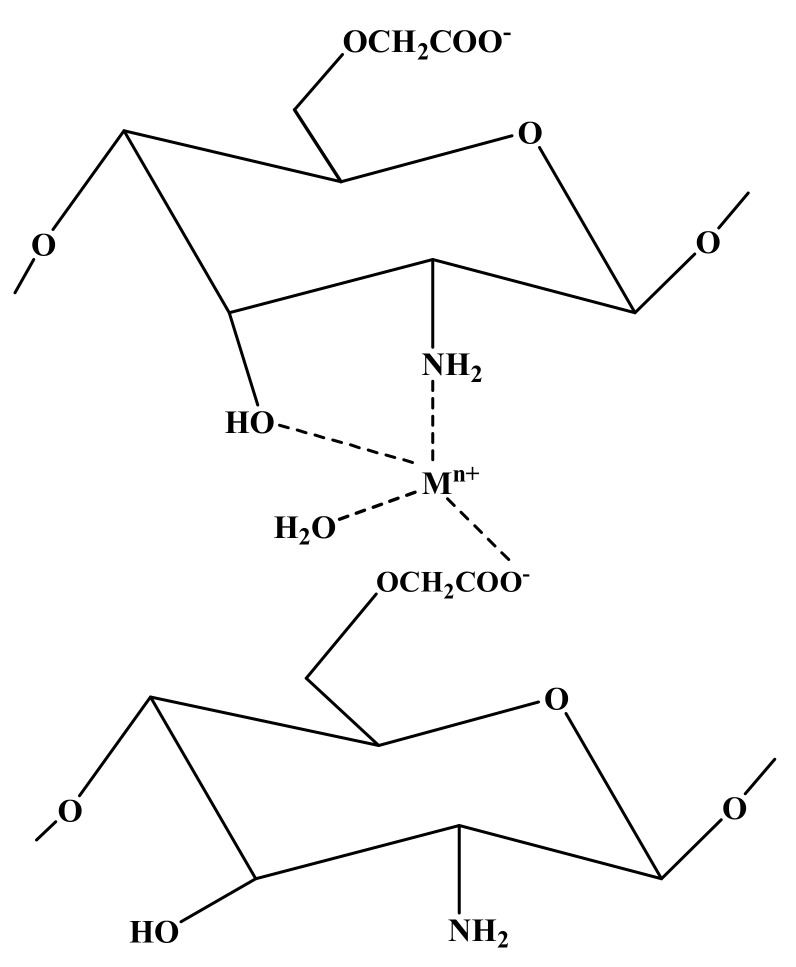
Cross-linking of carboxymethyl chitosan (CMCh) by metal ions [85].

Other metal ions like Cu^2+^ and Zn^2+^ can also have antimicrobial activity. Some antimicrobial hydrogels containing these metal ions have also been reported. For instance, Klinkajon and Supaphol [84] synthesized alginate hydrogels cross-linked by copper (II) using a two-step procedure. In the first step, alginate film was developed by the solvent-casting method using alginate solution that had been slightly cross-linked by copper (II) ions. In the second step, the films were further cross-linked by dipping in corresponding Cu^2+^ solution to improve their dimensional stability. Alginate solution (2% *w*/*v*) with copper sulfate solution (2% *w*/*v*) at low pH provided a soft film with the highest swelling ability. Increasing either the copper concentration or reaction time lead to a densely-cross-linked network with low water absorption capacity. The hydrogels were tested against *S. aureus*, *E. coli*, methicillin-resistant *S. aureus* (MRSA), *S. pyogenes* and *S. epidermidis*. The results showed excellent antibacterial activity against all kinds of bacteria, which depended on the concentration of copper (II) ions. Blood coagulation tests suggested that copper (II) cross-linked alginate hydrogels had an affinity to coagulate fibrin and probably had an effect on platelet activation and on pro-thrombotic coagulation. The authors claimed that the prepared films could be used as antibacterial candidates for wound dressings [84]. Recently, we have also reported carboxymethyl chitosan (CMCh) hydrogels cross-linked by metal ions (Ag^+^, Cu^2+^ and Zn^2+^) [85]. The hydrogelation process was fast and facile, i.e., the hydrogels were formed simply by mixing the solutions of CMCh and metal ions at an appropriate pH value, and the hydrogels formed within a few seconds. These hydrogels showed flexibility, elasticity, moldability and shape-persistent properties. The antibacterial properties were studied against *S. aureus* and *E. coli*, and the results showed excellent antibacterial properties against both kinds of bacteria. Figure 3 shows the proposed structure of CMCh with metal ions.

From the above examples, we may conclude that the hydrogels cross-linked by metal ions can enhance the antimicrobial activity, that a faster hydrogelation could be achieved, that the hydrogels may show self-healing properties and that the resultant hydrogels may show viscoelasticity. However, for biomedical applications, there is still a need to study the biocompatibility of metal ion-based hydrogels more.

## 6. Antimicrobial Hydrogels Containing Metal Nanoparticles 

### 6.1. Antimicrobial Hydrogels Containing Silver Nanoparticles 

Silver nanoparticles (Ag-NPs) are the most popular inorganic nanoparticles used as antimicrobial agents. The use of silver as an antimicrobial agent dates back centuries, when a solution of silver nitrate was commonly used for the treatment of illness. Even if silver has antimicrobial properties, it can also cause necrosis and apoptosis in mammalian cells [25]. Ag-NPs are widely used in textile products, injection molded plastics and for coating-based usages [86]. Ag-NPs are also used for biomedical applications [87]. It has been reported that Ag-NPs show higher antimicrobial properties than in the ionic form [88]. It has also been revealed that Ag-NPs show bactericidal activity against drug-resistant bacteria [89]. The antibacterial activity of Ag-NPs is due to the damage of the bacterial cell membrane [90]. Other researchers suppose that Ag-NPs produce pits and gaps in the bacterial membrane and then fragment the cell [91,92]. 

To control bacterial growth on surfaces, there is an increasing research interest to develop polymer-supported silver to use as antimicrobial consumer products. Recently, silver nanocomposite hydrogels for wound dressing and antimicrobial applications have been widely researched [25]. Yadollahi et al. [93] developed hydrogel nanocomposites by combining carboxymethyl cellulose (CMC), layered double hydroxide (LDH) and Ag-NPs. CMC-LDH/Ag nanocomposite hydrogels were prepared by the in situ formation of Ag-NPs in CMC-LDH. The resulting nanocomposite hydrogels were tested against *S. aureus* and *E. coli*, which showed antibacterial properties against both kinds of bacteria. Panacek et al. [94] prepared methyl cellulose/Ag nanocomposite hydrogels for topical antimicrobial applications. The prepared nanocomposite hydrogel showed antimicrobial activity against MRSA and yeast. The authors suggested that such nanocomposite hydrogels could represent a promising antimicrobial formulation for the treatment of wounds. In another study, chitosan hydrogel was prepared that was loaded with Ag-NPs as anti-biofilm materials [95]. The cytotoxicity of the nanocomposite hydrogel was also investigated against human fibroblast cells. The anti-biofilm activity of the nanocomposite hydrogel showed a log-reduction of 6.0 for the MRSA strain when the concentration of Ag-NPs was 100 ppm; while increasing the concentration of Ag-NPs to 1000 ppm, the log-reduction was 3.3. The biocompatibility evaluation on primary fibroblasts cells showed better results when the chitosan/Ag-NPs nanocomposite hydrogel was tested even with a high concentration of Ag-NPs. The authors concluded that chitosan/Ag-NPs nanocomposite hydrogels effectively prevented the development of biofilm and inhibited bacteria in established biofilm, which suggested that chitosan hydrogel with Ag-NPs could be applied in the treatment of chronic wounds. Another study was carried out to prepare acrylate-based silver nanocomposite hydrogels for bone graft applications [96]. Ag-NPs in the polymeric biomaterials provided non-antibiotic bactericidal activity against *S. epidermidis* and MRSA. Ag-NPs were incorporated into polymeric hydrogel by three different methods, i.e., entrapment into hydrogel before mineralization, adsorption after mineralization and diffusion during the crystallization of calcium phosphate. The adsorption method was the most effective method for the encapsulation, yet the adsorption of Ag-NPs inside the biomaterial caused a decrease in the antibacterial activity. 

**Figure 4:**
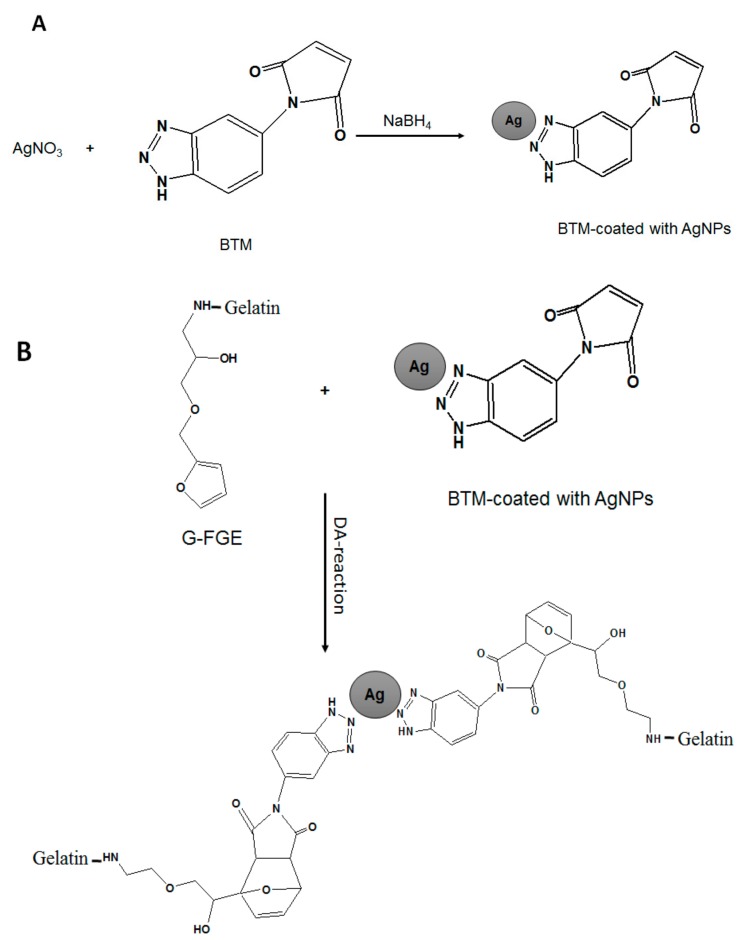
(**A**) Synthesis of coated BTM with Ag-NPs; (**B**) cross-linking of furan-modified gelatin (G-FGE) with Ag nanoparticle-coated BMT by the Diels–Alder cycloaddition reaction [97].

The biocompatibility and biodegradability are essential properties of materials for biomedical applications; therefore, Gabilondo et al. [97] reported a biocompatible hydrogel based on benzotriazole maleimide (BTM) and gelatin containing covalently-bound Ag-NPs. Maleimide was coated with Ag-NPs, and then, it was used as a cross-linker via Diels−Alder cycloaddition to furan-modified gelatin (gelatin-furfuryl glycidyl ether, i.e., G-FGE). The role of the Ag-NPs as cross-linkers for gelatin was confirmed by swelling and rheological measurements. The prepared nanocomposite hydrogels were tested for drug releasing and cytotoxicity. The nanocomposite hydrogel displayed potential for designing drug delivery system with low cytotoxicity, which could be used for a number of potential biomedical applications. Figure 4 shows a schematic representation of the loading of Ag-NPs into maleimide and cross-linking silver-coated maleimide with furan-modified gelatin to form the hydrogel. There are a number of polymeric hydrogels impregnated with Ag-NPs for a wide range of applications such as wound dressings, contact lenses, antimicrobial, biomedical and pharmaceutical fields. Some of them are tabulated in Table 1.

### 6.2. Antimicrobial Hydrogels Containing Other Metal Nanoparticles 

Although the majority of metal nanocomposite hydrogels for antimicrobial application studies involve silver nanoparticles, some other metals such as gold and copper nanoparticles have also been reported to form antimicrobial nanocomposite hydrogels. For instance, Marsich et al. [124] described a 3D hydrogel consisting of polysaccharides loaded with Au nanoparticles to assess their antibacterial activity. The results showed that the nanocomposite hydrogels were effective at inhibiting both Gram-positive and Gram-negative bacteria. However, the high cost of gold prevents the adoption of Au nanoparticles for such applications. Copper (Cu) is considered as a cost-effective substitute for Ag and Au nanoparticles for antimicrobial activity. Cometa et al. [125] have developed a poly(ethylene glycol diacrylate) hydrogel nanocomposite containing Cu nanoparticles (Cu-NPs); where the nanocomposite showed effective antibacterial activity, because of the charged quaternary ammonium salt and high surface/volume ratio of Cu-NPs. Recently, Villanueva and co-workers [126] developed a starch-based hydrogel containing Cu-NPs coated with silica. Cu-NPs were prepared using a copper salt solution followed by reduction with hydrazine. In order to improve the stability of Cu-NPs, a starch medium was used during the reduction followed by the silica-coating method. The starch hydrogels were prepared using water and urea as plasticizers and were imbibed with different concentrations of Cu-NPs coated with silica. The particle size was polydispersed, and the structure of the gel was changed along with the particle concentration. Larger pores were observed with higher concentrations of Cu-NPs. Antibacterial tests were carried out against *S. aureus* and *E. coli* bacterial species. The hydrogels were revealed to sustain the bactericidal activity for at least four cycles of use. A dermal acute toxicity test showed a slight irritation, proving the biocompatibility of the nanocomposite material. 

## 7. Antimicrobial Hydrogels Containing Metal Oxide Nanoparticles

Metal oxide nanoparticles form a new class of materials that is being progressively developed in research and health-related spheres. The usage of metal nanoparticles in practical fields requires their entrapment into natural or synthetic polymer matrices, so the resultant polymer/metal nanocomposite can be used per the requirement. Recently, metal oxide nanocomposite hydrogels have been demonstrated for their semi-conducting, ferromagnetic, UV-protecting and antimicrobial activities [127,128,129]. In this section, we will focus on antimicrobial metal oxide nanocomposite hydrogels.

### 7.1. Antimicrobial Hydrogels Containing ZnO Nanoparticles

The several positive characteristics of zinc oxide nanoparticles (ZnO-NPs) such as physiochemical stability, semiconductor behavior, photocatalytic activity, UV and infrared absorbing nature, antibacterial and non-toxic nature towards human make them valuable in different applications [130,131,132,133]. ZnO-NPs are effective at inhibiting both Gram-positive and Gram-negative bacteria. They are also effective against spores that show resistance at high pressure and temperature. The better bactericidal activity of ZnO-NPs compared to its microparticles is related to increased surface area [47,134]. The bactericidal efficacy of ZnO-NPs increases with the decrease of particle size [132]. Although ZnO-NPs reduce the bacterial viability, still their antibacterial mechanism is not well-understood so far. A proposed possibility is the production of hydrogen peroxide as the main factor for antibacterial activity. Another possibility is the accommodation of ZnO-NPs on the surface of the bacterial cell, which could damage the cell membrane and cause the death of the bacterial cell [135]. In addition, ROS generation on nanoparticles’ surfaces, nanoparticles’ internalization and zinc ion release could also be taken into account as the possible reasons for cell damage [40].

There have been great efforts to formulate ZnO-NPs/polymer nanocomposite hydrogels for several biomedical applications. The advantages of combining ZnO-NPs with hydrogel reside in its low cost and UV-blocking ability. Jayakumar et al. [136,137] have reported β-chitin/ZnO antibacterial nanocomposite hydrogels for wound dressing. The nanocomposite hydrogel was prepared by adding ZnO-NPs into the solution of β-chitin and then was freeze dried to obtain porous nanocomposite hydrogel. The nanocomposite hydrogel showed controlled swelling and degradation. The composite hydrogel demonstrated higher blood clotting capability and activation of platelets than the control. The antibacterial activity was investigated against *S. aureus* and *E. coli*, which exhibited bactericidal properties. The cytocompatibility of the nanocomposite hydrogel was evaluated using human dermal fibroblast cells (HDF), and these cells were viable on hydrogel composites similar to as on Kaltostat control bandage and pure β-chitin-based hydrogel. At higher concentrations of ZnO-NPs, the cells’ viability was 50–60%, while at lower concentrations, the viability was 80–90%. Faster healing and collagen deposition were observed in the in vivo evaluation of Sprague Dawley rats (S.D. rats) compared to the control. The authors concluded that the prepared hydrogels could be used for healing of wounds having a large volume of exudates. Similarly, collagen-dextran-ZnO-NPs nanocomposites as wound dressing material have been reported recently [138]. Nanocomposite hydrogels consisting of collagen and dextran with 50% ZnO-NPs were considered the most promising for future applications in wound dressing and a material with high potential in skin regeneration.

In another study, CMC was cross-linked by maleic acid, succinic acid and citric acid, and ZnO-NPs were loaded through in situ formation of ZnO-NPs in the hydrogel matrix. CMC hydrogel with excellent swelling was obtained by cross-linking using succinic acid (0.5%). The loading of ZnO-NPs into hydrogel resulted in antibacterial activity against both Gram-positive and Gram-negative bacterial strains [139]. Yadolahi et al. [140] used epichlorohydrin to cross-link CMC and then loaded ZnO-NPs in the hydrogel matrix for in situ formation. The swelling ability of the resulting nanocomposite hydrogel was investigated at different pH and salt solutions. Results showed the pH- and salt-sensitive swelling behavior of nanocomposite hydrogels. The antibacterial tests were carried out against *S. aureus* and *E. coli*, and the results showed the bactericidal effect of nanocomposite hydrogels. More recently, a flexible nanocomposite hydrogel film was developed by the combination of ZnO-NPs impregnated with mesoporous silica (ZnO-MCM-41) as a nano-drug carrier with CMC hydrogel [141]. To avoid the cytotoxicity of conventional cross-linking agents, citric acid was used as the cross-linker. CMC/ZnO-MCM-41 nanocomposite hydrogel showed high tensile strength, swelling ability and gas permeability. The drug delivery and bactericidal effect of nanocomposite hydrogel films were examined using tetracycline (TC), and the results showed a sustained TC release and excellent antibacterial properties against *S. aureus* and *E. coli*. The nanocomposite hydrogels showed cytocompatibility against adipose tissue-derived stem cells (ADSCs). Considering these properties, CMC/ZnO/MCM-41 could serve as a kind of promising wound dressing with sustained drug delivery properties. 

We have also reported CMCh/ZnO nanocomposite hydrogels for antibacterial applications [142]. CMCh was first cross-linked by epichlorohydrin, and then, ZnO-NPs were loaded via in situ synthesis of ZnO-NPs in the hydrogel network. The swelling of nanocomposite hydrogel was studied at different pH solutions. The maximum swelling was observed at pH = 7, while the nanocomposite hydrogel showed greater swelling in comparison to pure hydrogel. Moreover, we have also studied the bactericidal activity of nanocomposite hydrogels against *S. aureus* and *E. coli*. The pure hydrogel exhibited poor antibacterial activity, while the nanocomposite hydrogels showed excellent bactericidal activity against both kinds of bacteria. The antibacterial activity was dependent on the concentration of ZnO-NPs, and the higher concentration of ZnO-NPs showed higher antibacterial activity. In addition, the time-dependent bactericidal effect of the nanocomposites was also studied. The results showed a 100% reduction of bacterial population within 4 h of treatment with nanocomposite hydrogel.

Apart from natural polymers, synthetic polymers/ZnO-NPs nanocomposites have also been reported for antimicrobial applications. For instance, poly(vinyl alcohol)/ZnO nanocomposite hydrogel has been reported for wound dressing applications [143]. The biocompatibility of nanocomposite hydrogel was ascertained by bovine serum albumin (blood protein) adsorption, anti-hemolytic activity and in vitro cytotoxicity tests. Jonas et al. [144] have reported poly(*N*-isopropylacrylamide) (PNIPAAm) hydrogel loaded with ZnO-NPs for antibacterial surface coating applications. Nanocomposite hydrogel was developed by mixing PNIPAAm with ZnO-NPs, followed by spin coating and photo-cross-linking. The optical properties of ZnO-NPs were not affected by the polymer matrix as confirmed by UV-Vis spectroscopy. The nanocomposite films showed antibacterial properties against *E. coli* for a ZnO concentration as low as ≈0.74 μg/cm^2^ (1.33 mmol·cm^−3^), which was determined by inductively-coupled plasma optical emission spectrometry. The coatings showed non-toxicity towards a mammalian cell line (NIH/3T3) at bactericidal loading of ZnO over an extended period of seven days. The results concluded that the prepared nanocomposite hydrogel could be applied as coatings for biomedical devices [144]. There are some more antimicrobial hydrogels of synthetic and natural polymers containing ZnO-NPs given in Table 2.

### 7.2. Antimicrobial Hydrogels Containing CuO Nanoparticles

**Figure 5:**
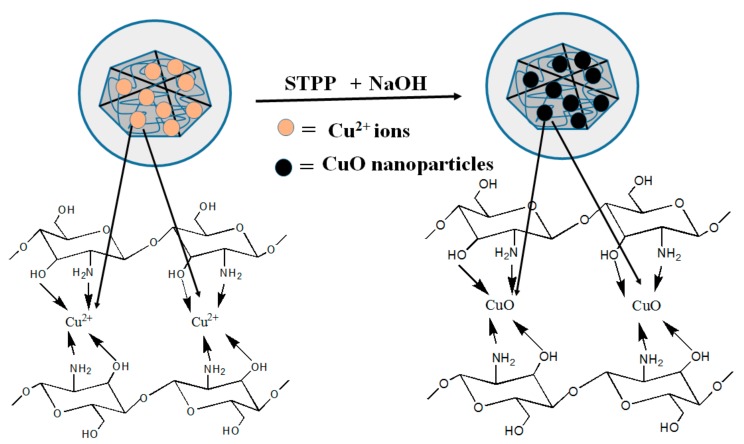
Schematic representation of chitosan with Cu^2+^ and CuO-NPs [159].

**Figure 6:**
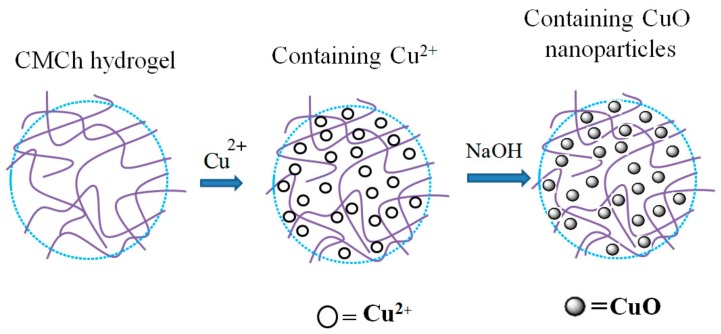
Schematic representation of incorporation of CuO-NPs into carboxymethyl chitosan hydrogel [160].

Copper oxide nanoparticles (CuO-NPs) are a brownish-black powder. They can be reduced to metallic copper on exposure to carbon monoxide or hydrogen at high temperature. CuO-NPs have been used for different applications such as catalysis, heat transfer fluids, gas sensors, solar cells and batteries [148]. CuO-NPs possess high photovoltaic and photocatalytic properties due to their narrowband gap in crystal structures [149]. Many research attempts have been carried out to investigate their antimicrobial properties. Ren et al. [150] studied the antimicrobial applications of CuO-NPs, and they observed the antimicrobial activity of CuO-NPs against a range of pathogens, including MRSA and *E. coli*. Meghana et al. [151] examined the mechanism of antimicrobial activity of CuO-NPs. Their conclusions strongly support the contact killing mechanism of CuO-NPs of bacterial cells. According to them, CuO-NPs cause bacterial cell lysis in two stages. In the first stage, CuO-NPs damage the bacterial cell, while in the second stage, CuO-NPs produce free radicals, which toxicate the inner cell complexes. Applerot and coworkers [46] reported the production of reactive oxygen species (ROS) by CuO-NPs adhered to bacterial cells, which in turn provoked an improvement of the intracellular oxidative stress. They used several methods such as lipid peroxidation and reporter strains of oxidative stress to confirm their results. They also carried out the electron microscopic study, which showed the penetration of small CuO-NPs inside the cells. One of the first CuO-based products was put forth by a private company, Cupron. The CuO micro-sized particles were incorporated into the polymer, which was further made into a wound dressing. These dressings prevented infection effectively and increased the rate of wound healing, compared to standard treatments [152]. Recently, a Cu-based nanocomposite has been reported with several polymers such as cellulose [153], chitosan [154], polyacrylic acid [155] and polypropylene [156]. The combination of CuO-NPs with polymers to form antimicrobial nanocomposite hydrogels is quite attractive. Various groups of researchers are working on antimicrobial CuO-based nanocomposite hydrogels. Recently, CMC-based hydrogels loaded with CuO-NPs have been reported by various research groups. Hebeish and Sharaf [157] developed a copolymeric hydrogel nanocomposite for wound dressing applications. They used diallyldimethylammonium chloride (DADMAC) monomer and *N*,*N*′-methylene-bis-acrylamide (MBA) cross-linking agent on water-soluble CMC. The in situ synthesis of CuO-NPs within the matrix of the CMC-DADMAC hydrogel nanocomposite attached to cotton fabric was studied. The antibacterial properties of the nanocomposite hydrogel were investigated against Gram-positive and Gram-negative bacteria. The nanocomposite hydrogel showed excellent antibacterial activity. Furthermore, they also loaded Ag-NPs to CMC-DADMAC hydrogel, which showed better antibacterial results than the CMC-DADMAC/CuO nanocomposite hydrogel. Another study was carried out to investigate the antibacterial properties of carboxymethyl cellulose (CMC) hydrogel loaded with CuO-NPs, and epichlorohydrin was used as the cross-linking agent for CMC [158]. CuO-NPs were synthesized inside the hydrogel matrix by the in situ method. The swelling behavior of the hydrogel nanocomposite was studied in different pH and salt solutions. The bactericidal activity of nanocomposite was investigated against *S. aureus* and *E. coli*. The results exhibited a bactericidal effect against both kinds of bacteria. The authors concluded the possible use of the nanocomposite hydrogel in the biomedical field. In addition to CMC, chitosan-based antibacterial hydrogels loaded with CuO-NPs have also been reported recently. Farhoudian et al. [159] prepared antibacterial chitosan/CuO nanocomposite hydrogel beads. Chitosan was cross-linked by sodium tripolyphosphate (STPP), while CuO-NPs were formed in situ during the formation of hydrogel beads (Figure 5). The particle size of CuO-NPs ranged from 10–25 nm. The prepared hydrogel showed pH-sensitive swelling behavior. The nanocomposite hydrogel beads showed antibacterial activity against *S. aureus* and *E. coli*. Recently, we have also reported CMCh/CuO-NPs nanocomposite hydrogels [160]. The cross-liking of CMCh was carried out by epichlorohydrin, and CuO-NPs were loaded in situ into the prepared hydrogel (Figure 6). The nanoparticle size ranged from 20–50 nm. The antibacterial activity of CMCh/CuO-NPs was tested against *S. aureus* and *E. coli*. The pure hydrogel did not show antibacterial activity, while the nanocomposite hydrogel showed CuO-NP-dependent antibacterial activity. 

Recently, carrageenan hydrogels were prepared and were loaded with ZnO-NPs and CuO-NPs separately, as well as collectively; and their various properties were studied, like swelling behavior, mechanical properties and antibacterial properties [161]. The neat hydrogel was transparent, but it became translucent when metal oxide nanoparticles were incorporated. The swelling ratio of carrageenan hydrogel was 2980%, while it was improved to 3535% after introduction of ZnO-NPs. The carrageenan-based hydrogel exhibited strong antibacterial activity against food-borne pathogenic bacteria, i.e., *E. coli* and *L. monocytogenes*. The ZnO-NP-incorporated hydrogel exhibited higher water holding capacity, mechanical strength, UV-screening, thermal stability and antibacterial properties compared to CuO-NP-incorporated ones. The nanocomposite hydrogel could have high potential for biomedical, cosmetic and active food packaging areas.

### 7.3. Antimicrobial Hydrogels Containing TiO_2_ Nanoparticles

The antimicrobial properties of TiO_2_ nanoparticles (TiO_2_-NPs) are related to its crystal size and shape [162]. It has been proposed that oxidative stress via the generation of ROS could be a possible mechanism for TiO_2_-NPs (anatase form). ROS can cause site-specific DNA damage of the bacterial cell [163]. Roy and coworkers [163] investigated the effect of TiO_2_-NPs with different antibiotics against MRSA. They found that the TiO_2_-NPs with different antibiotics improved the antibacterial effect of cephalosporins, aminoglycosides, macrolides, beta lactams, glycopeptides, lincosamides and tetracycline against MRSA. The photocatalytic activity of TiO_2_-NPs helps them with the complete eradication of bacteria. In fact, TiO_2_-NPs produce ROS under UV light. The antibacterial photocatalytic property is accompanied by lipid peroxidation that causes improved membrane fluidity and interrupts the cell integrity [164]. The usage of TiO_2_-NPs under UV light is limited because of the damage to human cells and tissues [89]. The doping of TiO_2_-NPs with metal ions could be a good idea to solve this problem [165]. The doped TiO_2_-NPs with metal ions shift the light absorption range to visible light, and therefore, there is no need to irradiate TiO_2_-NPs with UV light. Another solution to the problem is to conjugate TiO_2_-NPs with non-toxic polymers [89]. 

Various studies showed the incorporation of TiO_2_-NPs into polymers, such as ethylene vinyl alcohol copolymer [166], polypropylene [167] and poly[2-(*tert*-butylamino) ethyl methacrylate-*co*-ethylene glycol dimethacrylate [168]. The resultant novel nanocomposites showed remarkable performance against Gram-positive and Gram-negative bacterial strains. Moreover, due to the highly active surface chemistry of TiO_2_-NPs, they tend to interact with polymers in which they are dispersed. Therefore, they effect the physical and chemical properties of nanocomposites. Zhang et al. [169] reported methacrylated gelatin hydrogel films with in situ-synthesized TiO_2_-NPs as a biomaterial. TiO_2_-NPs were homogeneously dispersed in hydrogel films with the size ranging from 85–130 nm depending on the concentration of TiO_2_-NPs. The water absorption capability of various nanocomposite hydrogel films was in the range of 471–758%, which could be suitable to prevent the accumulation of exudates on wound beds. The antibacterial activity of the nanocomposite hydrogel films was evaluated against *S. aureus* and *E. coli* by the shake flask test. The results demonstrated the good performance of nanocomposite hydrogel films. Cytotoxicity tests showed that all films were nontoxic and revealed favorable adherence in the presence of L929 cells. Thus, the nanocomposite hydrogels could have potential applications as antibacterial wound dressing materials. 

Recently, photosensitive antibacterial TiO_2_-NPs/CMCh/PVA ternary nanocomposite hydrogels have been reported [170]. Nanocomposite hydrogels were prepared by freezing-thawing cycles and electron-beam radiation with PVA, CMCh and TiO_2_-NPs as raw materials. The presence of TiO_2_-NPs in the hydrogel matrix was confirmed by thermogravimetry, X-ray powder diffraction and field emission scanning electron microscopy. The microscopic study revealed a porous structure of TiO_2_/CMCh/PVA nanocomposite hydrogel, while at the same time, the nanoparticles were observed inside the hydrogel matrix. In addition, the nanocomposite hydrogel showed significant antibacterial activity against *E. coli* and *S. aureus* as model bacteria. The antibacterial activity was demonstrated by the plate count method, circle method and cell density method. The cytotoxicity test of the nanocomposite hydrogels was carried out against L929 cells. The results suggested no obvious cytotoxicity of TiO_2_/CMCh/PVA nanocomposite hydrogel. The developed hydrogel could be a biocompatible, photosensitive and antibacterial material for various applications like cosmetics, medical dressings and environmental protection. 

### 7.4. Antimicrobial Hydrogels Containing MgO Nanoparticles

The antibacterial mechanism of MgO nanoparticles (MgO-NPs) brings about the production of superoxide on the surface of these particles and also a high pH value due to the hydration of MgO-NPs by water [171]. It is also reported that MgO-NPs damage the cell membrane, which in turn leads to the death of bacterial cells [172]. The existence of active oxygen specie, e.g., superoxide on the surface of MgO-NPs, is one of the major factors affecting its bactericidal activity [173]. Low cost, biocompatibility and availability of MgO-NPs make them favorable for antibacterial applications [41]. Scientists believe that these materials can be applied in environmental protection, food processing and medical treatment [27]. However, there is little work reported about the nanocomposite hydrogels containing MgO-NPs. For instance, Supriyanto et al. [174] reported a polyacrylamide/MgO nanocomposite hydrogel. Hydrogel was synthesized using sodium carboxymethylcellulose (NaCMC); *N*,*N*′-methylenebisacrylamide (MBA) was used as the cross-linking agent, and ammonium persulfate (APS) and *N*,*N*,*N*′,*N*′-tetramethylethylenediamine (TEMED) were used as the initiator. MgO-NPs were added to the hydrogel matrix to examine the antibacterial properties of the nanocomposite hydrogels. The characterization of the hydrogel gave valuable information about the structure of the polymer, the swelling behavior and the bonding formation of the hydrogel. The incorporation of NaCMC into the hydrogel enhanced its physical and chemical properties, like swelling capacity, strength and flexibility. The antibacterial test was carried out against *E. coli* through the agar diffusion method. The pure hydrogel showed a lower inhibition zone towards *E. coli*. However, by the addition of 0.3 g of MgO-NPs to the hydrogel, the antibacterial properties were enhanced three times compared to the pure hydrogel. 

## 8. Conclusions

Due to an increasing microbial resistance to common antibiotics and disinfectants, much investigation has been carried out to produce antimicrobial materials. Metals and metal oxide nanocomposite hydrogels could be considered as appropriate alternatives for some antimicrobial applications. We have reviewed such nanocomposite hydrogels as an emerging class of materials appropriate for antimicrobial and wound dressing applications. We expect that the summarization of nanocomposite hydrogels in this review delivers a better understanding of the system and assists the readers with the design of innovative combinations for new applications. In the future, such a design of nanocomposite hydrogels will lead to advanced applications, as well as the navigation of the fundamental understanding of material interactions.

## Figures and Tables

**Table 1:** Antibacterial silver nanocomposite hydrogels.

Nanocomposite Hydrogel	Loading of Ag Nanoparticles	Antimicrobial Activity against	Applications	Ref.
Poly(vinyl alcohol)/sodium alginate/silver	Entrapment during physical cross-linking	*E. coli*	Food packaging	[98]
Almond gum-poly(acrylamide)/Ag	In situ preparation of Ag-NPs in hydrogel	*S. aureus, E. coli, P. aeroginosa*	Antibacterial material	[99]
Acrylic acid/Ag	UV-irradiation	*S. aureus, C. albicans, P. aeruginosa, S. epidermidis, E. coli, A. baumannii, K. pneumonia*	Super absorbent antimicrobial material for pharmaceutical applications	[17]
Kappa-Carrageenan (κ-Carrageenan)/Ag	Biosynthesis of Ag-NPs in *Citrullus colocynthis* seed extract	*S. aureus*, MRSA, *P. aeruginosa, E. coli*	Pharmaceutical applications	[26]
Carboxymethyl cellulose/Ag	In situ preparation of Ag-NPs with synthesis of hydrogel.	*S. aureus K. pneumonia*	Treatment of cotton fabrics for medical applications	[100]
*Tragacanth gum*/Ag	In situ of nano silver in hydrogel	*S. aureus, E. coli*	For cotton fabrics to improve its properties	[101]
Polyvinyl alcohol/Ag	Loading of Ag-NPs to polymeric hydrogel	*S. aureus, E.coli, P. aeruginosa*	Antimicrobial dressing scaffold	[102]
Silver/starch/polyacrylamide nanocomposites	In situ preparation of Ag-NPs inside hydrogel network	*S. aureus, E. coli, A. flavus, C. albicans*	Antimicrobial applications	[103]
Chitosan based hydrogel/Ag nanocomposites	UV-radiation	*S. aureus, E. faecalis*	Biomedical applications	[104]
Polysaccharides such as xanthan gum (XG) and chitosan (CS)/Ag nanocomposite hydrogel	In situ formation of Ag-NPs within hydrogel.	*S. aureus, E. coli*	Antibacterial wound dressing	[105]
Tragacanth gum and graphene oxide/Ag	In situ reduction of Ag^+^ to Ag^0^ flower extract of *Achillea millefolium*	*S. aureus*	Bio-absorbent for removal of heavy metals from water, with antimicrobial activity.	[106]
Carboxymethyl chitosan/Ag nanocomposite hydrogel	Synthesis of Ag-NPs with the formation of hydrogel.	*S. aureus, S. faecalis, B. subtilis, P. aeruginosa, E.coli, N. gonorrhoeae, C. albicans*	Antimicrobial hydrogels	[107]
Silicone/Ag nanocomposite hydrogel film	In situ chemical reduction of Ag^+^ by NaBH_4_	*B. subtilis, S. aureus, E. coli, P. aeruginosa*	Contact lenses	[108]
Carboxymethyl cellulose (CMC), polyvinyl alcohol (PVA)/Ag	Incorporation of Ag-NPs by microwave radiations	*E. coli, P. aeruginosa, K. pneumoniae, P. vulgaris, S. aureus P. mirabilis*	Antibacterial applications	[109]
Poly (methyl methacrylate-*co*-acryloyl phenylalanine) (PMAPA)/Ag	Chemical reduction of Ag^+^ by NaBH_4_	*Bacilli, E. coli*	Antibacterial applications	[110]
Iota-Carrageenan/Ag nanocomposite hydrogel	Biochemical reduction of Ag^+^ by leaf extract of *Azadirachta indica*	*Bacillus, E. coli*	Antimicrobial applications	[111]
Silver/poly(vinyl alcohol)/graphene nanocomposite hydrogel film	Electrochemical reduction of Ag^+^ ions.	*E. coli, S. aureus*	Wound dressing	[112]
Polythioether dendron/Ag	In situ chemical reduction of Ag^+^ by NaBH_4_	*E. coli* and anti-algae	Antifouling coating of biomaterials.	[113]
Carboxymethyl cellulose/Ag nanocomposite hydrogel	In situ preparation of Ag-NPs in hydrogel as well as incorporation of Ag-NPs into hydrogel	*P. aeruginosa, E. coli, S. aureus, B. subtilis.*	Antibacterial applications	[114]
Ag-poly(*N*-isopropylacrylamide/itaconic acid) hydrogel	In situ reduction of Ag^+^ with gamma irradiation	*S. aureus, E. coli*	Antibacterial applications	[115]
Gum acacia (GA)/poly(sodium acrylate)/Ag semi-interpenetrating polymeric hydrogel	In situ reduction of Ag^+^ with extract of *Syzygium aromaticum* (clove)	*E. coli*	Antibacterial applications	[116]
Ag/Alginate nanocomposite hydrogel	Electrochemical production of Ag-NPs in hydrogel.	*S. aureus, E. coli*	Wound dressing	[117]
Chitosan-polyethylene glycol/Ag nanocomposite hydrogel	In situ reduction of Ag^+^ as well as incorporation of Ag-NPs into hydrogel	*E. coli*	Antimicrobial	[118]
Tea/polyacrylamide/Ag nanocomposite hydrogel	In situ reduction of Ag^+^ by mint leaf extract within hydrogel	*S. aureus, E. coli*	Antimicrobial	[119]
Alginate/polyvinyl alcohol/ poly(*N*-vinyl-2-pyrrolidone)/ Ag nanocomposite hydrogel	Electrochemical synthesis of Ag-NPs in hydrogel	*E. coli*	Wound dressing	[120]
Poly(sulfobetaine acrylamide)/Ag	In situ formation of Ag-NPs within hydrogel	*S. epidermidis, P. aeruginosa*	Treatment of infected chronic wounds	[121]
2-hydroxyethyl methacrylate/itaconic acid/Ag	In situ reduction of silver nitrate by gamma radiations	*E. coli, S. aureus, C. albicans*	Wound dressing	[122]
Poly(acryl amide-co-acryloyl phenyl alanine)/Ag	In situ chemical reduction of Ag^+^ by NaBH_4_	*S. aureus, E. coli*	Artificial burn dressing	[123]

**Table 2:** Antimicrobial hydrogels loaded with ZnO nanoparticles.

Nanocomposite Hydrogel	Loading of ZnO Nanoparticles	Antimicrobial Activity against	Applications	Ref.
Gum acacia/poly sodium acrylate/ZnO	In situ preparation by hydrothermal method	*E. coli*	Antimicrobial	[64]
Alginate/gum acacia/ZnO	Addition of ZnO-NPs during the synthesis of hydrogel	*P. aeruginosa, B. cereus*	Wound dressing	[19]
Poly(ethylene glycol) methyl ether methacrylate modified ZnO (ZnO-PEGMA)/4-azidobenzoic agarose (AG-N3) IPN hydrogel	Addition of ZnO-NPs to PEGMA	*S. aureus, E. coli*	Wound dressing	[145]
Chitosan/ZnO flexible, microporous hydrogel	Incorporation of ZnO-NPs into hydrogel	*S. aureus, E.coli*	Wound dressing	[146]
Genipin-crosslinked chitosan (GC), poly(ethylene glycol) (PEG)/ZnO/Ag	Incorporation of ZnO and Ag nanoparticles into hydrogel matrix	*E. coli, P. aeruginosa, S. aureus, B. subtilis*	Wound dressing	[147]

## Abbreviations

*A. baumannii*	*Acinetobacter baumannii*
*A. flavus*	*Aspergillus flavus*
ADSCs	Adipose tissue-derived stem cells
Ag-NPs	Silver nanoparticles
APS	Ammonium persulfate
ATRP	Atom transfer radical polymerization
*B. subtilis*	*Bacillus subtilis*
BTM	Benzotriazole maleimide
*C. albicans*	*Candida albicans*
CMC	Carboxymethyl cellulose
CMCh	Carboxymethyl chitosan
Cu-NPs	Copper nanoparticles
CuO-NPs	Copper oxide nanoparticles
DADMAC	Diallyldimethylammonium chloride
DAM	Dopamine methacrylamide
*E. coli*	*Escherichia coli*
*E. faecalis*	*Enterococcus faecalis*
EDTA	Ethylene diamine tetraacetic acid
GA	Gum acacia
HDF	Human dermal fibroblast
*K. pneumonia*	*Klebsiella pneumoniae*
*L. monocytogenes*	*Listeria monocytogenes*
LDH	Layered double hydroxide
MBA	N,N′-methylene-bis-acrylamide
MgO-NPs	Magnesium oxide nanoparticles
MRSA	Methicillin-resistant *Staphylococcus aureus*
*N. gonorrhoeae*	*Neisseria gonorrhoeae*
*P. aeruginosa*	*Pseudomonas aeruginosa*
PEG	Poly(ethylene glycol)
PEGMA	Poly(ethylene glycol) methyl ether methacrylate
PEIs	Poly(ethyleneimine)s
PMAPA	Poly (methyl methacrylate-*co*-acryloyl phenylalanine)
PNIPAAm	Poly(N-isopropyl acrylamide)
PVA	Poly vinyl alcohol
RAFT	Reversible addition fragmentation chain-transfer polymerization
ROP	Ring-opening polymerization
ROS	Reactive oxygen species
*S. aureus*	*Staphylococcus aureus*
*S. epidermidis*	*Staphylococcus epidermidis*
*S. pyogenes*	*Streptococcus pyogenes*
S.D. rats	Sprague-Dawley rats
SBE	Sulfobetaine
TEMED	*N*,*N*,*N*′,*N*′-tetramethylethylenediamine
TiO_2_-NPs	Titanium dioxide nanoparticles
TTO	Tea tree oil
XG	Xanthan gum
ZnO-NPs	Zinc oxide nanoparticles
